# Identification and Analysis of the SET-Domain Family in Silkworm, *Bombyx mori*

**DOI:** 10.1155/2015/161287

**Published:** 2015-10-19

**Authors:** Hailong Zhao, Chunqin Zheng, Hongjuan Cui

**Affiliations:** State Key Laboratory of Silkworm Genome Biology, Southwest University, Chongqing 400716, China

## Abstract

As an important economic insect, *Bombyx mori* is also a useful model organism for lepidopteran insect. SET-domain-containing proteins belong to a group of enzymes named after a common domain that utilizes the cofactor S-adenosyl-L-methionine (SAM) to achieve methylation of its substrates. Many SET-domain-containing proteins have been shown to display catalytic activity towards particular lysine residues on histones, but emerging evidence also indicates that various nonhistone proteins are specifically targeted by this clade of enzymes. To explore their diverse functions of SET-domain superfamily in insect, we identified, cloned, and analyzed the SET-domains proteins in silkworm, *Bombyx mori*. Firstly, 24 genes containing SET domain from silkworm genome were characterized and 17 of them belonged to six subfamilies of SUV39, SET1, SET2, SUV4-20, EZ, and SMYD. Secondly, SET domains of silkworm SET-domain family were intraspecifically and interspecifically conserved, especially for the catalytic core “NHSC” motif, substrate binding site, and catalytic site in the SET domain. Lastly, further analyses indicated that silkworm SET-domain gene BmSu(var)3-9 owned different characterization and expression profiles compared to other invertebrates. Overall, our results provide a new insight into the functional and evolutionary features of SET-domain family.

## 1. Introduction

SET-domain superfamily includes all but one of the methyltransferases that methylate specific site of histone lysine (K) residues involved in epigenetic regulation. This family is characterized by the highly conserved SET (Su(var)3-9, E(z), Trithorax) domain that consists of approximately 130 amino acids and is responsible for the catalytic activity of these methyltransferases. Through transferring a methyl group from S-adenosyl-L-methionine (AdoMet) to the amino group of a lysine residue on different sites of histone proteins, SET-domain methyltransferases function in making different histone methylation marks. Histone methylation is very important for the chromatin modification and regulation of gene expression [1–4], which plays a crucial role in the animal development and a number of other biological processes, such as heterochromatin establishment, transcription regulation, parental imprinting, and cell fate destination. Researches also suggest that SET-domain proteins are closely related to many human diseases [5–10].

Based on the substrate specificity towards histones, SET-domain-containing lysine methyltransferases can be classified into subgroups like KMT1 (H3K9), KMT2 (H3K4), KMT3 (H3K36), KMT4 (H3K79), KMT5 (H4K20), and KMT6 (H3K27) methyltransferases [11]. SET-domain proteins can be divided into seven main subfamilies (SUV39, SET1, SET2, EZ, SUV4-20, SMYD, and RIZ families) as well as some unclassified members like SET7/9 and SET8. Members in each subfamily have very high similarity not only in the SET-domain amino acid sequence but also in the flank motifs of SET domain.

Members of this family have been studied extensively for their function in modifying histones by methylating them directly and thus changing the mode of chromatin to regulate the binding of cofactors in many research models, such as human, mouse,* Drosophila*,* Arabidopsis*, and yeast. On the other hand, more and more recent studies give sufficient evidence of the fact that SET-domain-containing proteins also regulate many nonhistone substrates, including some transcriptional factors and tumor suppressors [12–17]. Epigenetic regulation of SET-domain family remains to be studied in lepidopteron insects. Based on silkworm genome database, we identified, cloned, and analyzed the SET-domains proteins in silkworm,* Bombyx mori*. Here, we provide an overview of the common and unique features of silkworm SET-domain family members, which will provide more information and important reference to the whole SET-domain proteins.

## 2. Materials and Methods

### 2.1. Silkworm

The silkworm strain, Dazao (p50), used in this study is maintained by the State Key Laboratory of Silkworm Genome Biology. The silkworm larvae were reared with fresh mulberry leaves under 25°C, with a 12 h/12 h photoperiod.

### 2.2. Datasets

We combined the updated Silkworm 9x genomic sequencing database, silkworm EST database, CDS database, and silkworm predicted protein database (all found at SilkDB, http://silkworm.swu.edu.cn/silkdb/). Gene sequences of mammals,* C. elegans*,* D. melanogaster*, and some other insects were downloaded from GenBank (http://www.ncbi.nlm.nih.gov/).

### 2.3. Screening of SET-Domain Family Genes

Protein sequences of SET-domain family members from other species were used to query the silkworm database with *E*-value less than 0.1. The hits in the screening were furthermore confirmed by blast in NCBI protein database. Besides, the conserved SET-domain sequences of other species were also used as query sequence to blast in silkworm database. Moreover, we used online protein domain prediction program SMART (http://smart.embl-heidelberg.de/) and Pfam (http://pfam.sanger.ac.uk/) to validate the SET domain in screening hits from silkworm database.

### 2.4. RNA Extraction

Different tissues of silkworm larvae were collected and stored in liquid nitrogen until use. Trizol reagent (Invitrogen, USA) was used to extract the total RNA of silkworm tissues. RNA concentration was calculated by spectrophotometer. RNA samples were digested by RNase-free DNase I (TaKaRa, Japan) to get rid of genomic DNA contamination. 2 *μ*g of each RNA sample was used to synthesize the first strand of cDNA by M-MLV Reverse Transcriptase following the manufacturer's instructions (Promega, USA).

### 2.5. Verification of Identified Genes and Expression Analysis

Primers were designed for most of the identified silkworm SET-domain family members to clone them from silkworm cDNA. Silkworm cytoplasmic gene Actin3 (forward primer: 5′-AAC ACC CCG TCC TGC TCA CTG-3′; reverse primer: 5′-GGG CGA GAC GTG TGA TTT CCT-3′) was used as an internal control. PCR amplification was performed using cDNA of deferent silkworm tissues to examine their expression profile. The 20 *μ*L PCR reaction volume is as follows: initial denaturation at 94°C for 3 min, followed by 30 cycles of 30 s at 94°C, annealing at temperatures *T*_*m*_ (usually set at 55°C) for 45 s and 1 min extension at 72°C, and extension at 72°C for 10 min. The PCR products were analyzed by 1% agarose gels.

### 2.6. Phylogenetic Analysis

SET-domain amino acid sequences of silkworm SET-domain family candidates were aligned to each other and also with the representative SET domains of other species by the program ClustalX. The sequences of silkworm SET-domain-containing proteins were applied to construct phylogenetic trees by neighbor-joining algorithm (1000 bootstrap replicates) with the program MEGA4.0.

## 3. Results and Discussion

### 3.1. Identification of Silkworm SET-Domain Family

We have identified the silkworm SET-domain family genes from silkworm database SilkDB for the first time. We have found 25 genes containing SET domain from silkworm genome by screening. Referring to the classification method of SET-domain family members in other species which is based on the SET-domain sequence and the feature of its flank motifs or domains, we were able to characterize 17 of them into six subfamilies of SUV39, SET1, SET2, SUV4-20, EZ, and SMYD (see Table 1). Silkworm SET-domain genes are mainly located on chromosomes 1, 3, 4, 15, 16, and 23, and 12 of them have ESTs in the database. We did not find any homologous genes of mammal RIZ subfamily in silkworm database; this may be the species difference like the SET proteins SET1, SET2 can be found in yeast while homolog genes of Su(var)3-9 and EZH1/2 are missing.

### 3.2. Cloning and Bioinformatics Analysis

**Figure 1:**
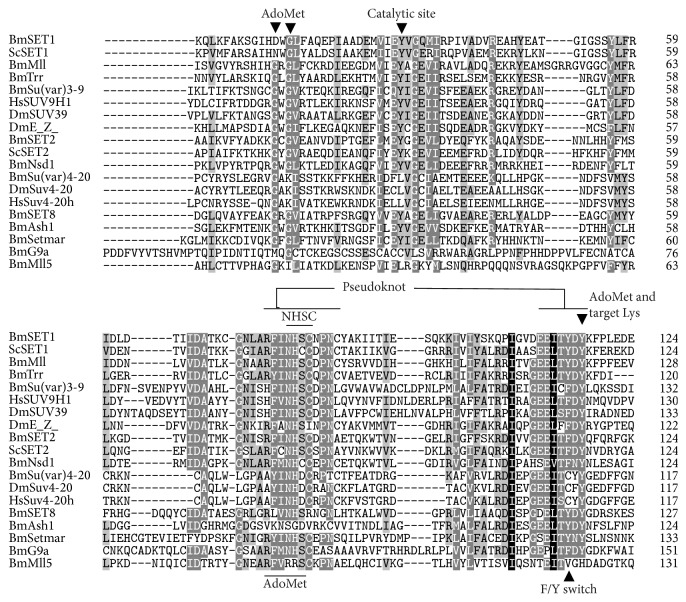
SET-domain sequence alignment analysis. The figure shows invariant residues in binding to the catalytic substrate AdoMet (S-adenosyl-L-methionine) the target lysine, catalytic site, the most conserved NHSC motif, F/Y switch controlling whether the product is a mono-, di-, or trimethylated histone, and the pseudoknot structure formed by two conserved SET motifs to form an active site in a location immediately next to the peptide-binding cleft. Sc:* Saccharomyces cerevisiae*; Hs:* Homo sapiens*; Dm:* Drosophila melanogaster*.

According to the gene sequence from silkworm genome database, we designed primers to clone and verify whether these genes are true hits. We have cloned most members of the silkworm SET-domain family (see Table 1). Through bioinformatics analysis, we found that SET domains of silkworm SET-domain family are intraspecifically and interspecifically conserved, especially for the catalytic core “NHSC” motif, substrate binding site, and catalytic site in the SET domain (Figure 1).

### 3.3. Phylogenetic Analysis of Silkworm SET-Domain Subfamilies

We have selected the representative subfamily to make phylogenetic analysis; we found that the domain architecture of SUV4-20 subfamily and SETDB1 and Su(var)3-9 of SUV39 subfamily is highly conserved to other species. The three members of silkworm SET2 subfamily are clustered to their homolog of other species, respectively.

#### 3.3.1. SUV4-20 Subfamily

**Figure 2:**
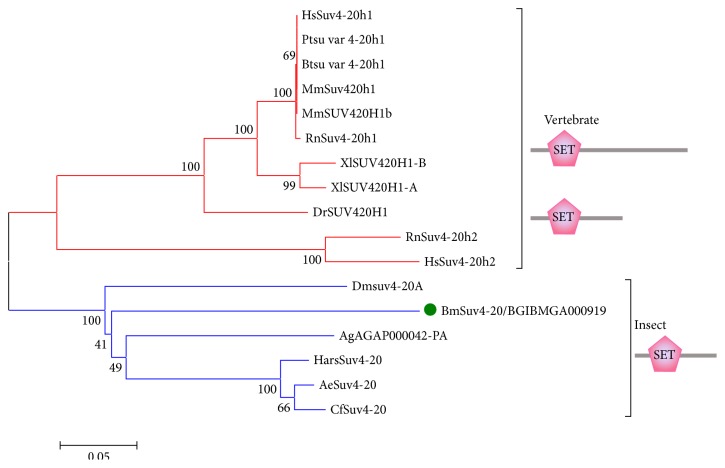
Phylogenetic analysis of SUV4-20 subfamily. Pt:* Pan troglodytes*; Mm:* Mus musculus*; Bt:* Bos taurus*; Rn:* Rattus norvegicus*; Xl:* Xenopus laevis*; Dr:* Danio rerio*; Ag:* Anopheles gambiae* str. PEST; Har:* Harpegnathos saltator*; Ae:* Acromyrmex echinatior*; Cf:* Camponotus floridanus* (the abbreviations of species mentioned above are omitted here).

SUV4-20 subfamily methylates H4K20 in other species. Human, mouse, and* Xenopus laevis* have two SUV4-20 members, while in* Drosophila* and several other insects there is only one member. In silkworm, we identified only one SUV4-20 member like other insects. Phylogenetic analysis of SUV4-20 subfamily suggests that BmSuv4-20 clusters with Suv4-20 proteins of* Drosophila*,* Anopheles gambiae*, and other insects (Figure 2). Besides, SUV4-20 proteins of vertebrates cluster together. Among vertebrates, Suv4-20h2 (Suv4-20 homolog 2) of human and* Rattus norvegicus* are shorter than Suv4-20h1 (Suv4-20 homolog 1) and cluster together. BmSuv4-20 has the same domain structure as SUV4-20 proteins of other species that there is only one domain the SET located on the N-terminal of them (all shown in Figure 2).

#### 3.3.2. SUV39 Subfamily

**Figure 3:**
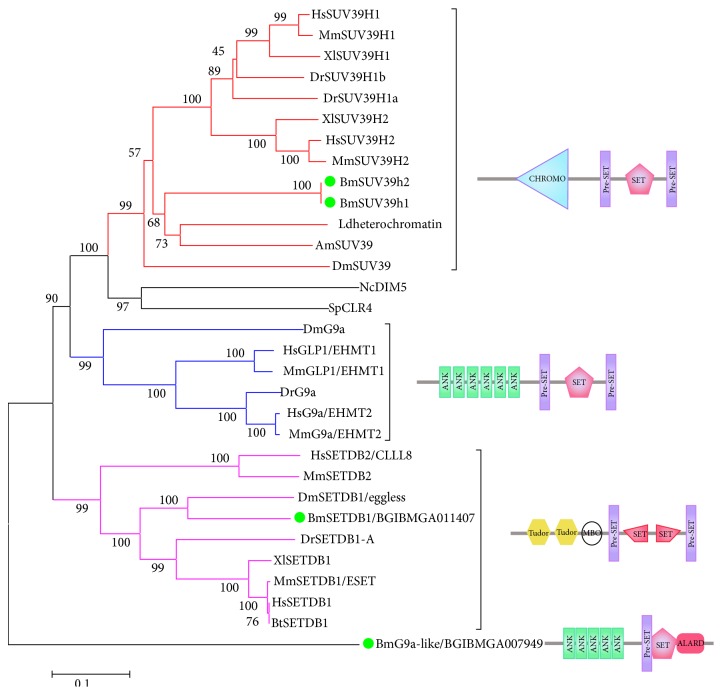
Phylogenetic analysis of SUV39 subfamily. Nc:* Neurospora crassa*; Sp:* Schizosaccharomyces pombe*; Am:* Apis mellifera*; Ld:* Leptinotarsa decemlineata*. Ldheterochromatin represents the heterochromatin protein of* Leptinotarsa decemlineata* SUV39 subfamily. DIM5 and CLR4 are representative SUV39 subfamily members of* Neurospora crassa* and* Schizosaccharomyces pombe*. The bright green on the phylogenetic tree represents silkworm members (the abbreviations of species mentioned above are omitted here). Domains: CHROMO (CHRromatin Organization MOdifier), Pre-SET (Cys-rich putative Zn^2+^-binding domain that occurs at N-terminal to some SET domains), and Post-SET (Cysteine-rich motif following a subset of SET domains).

**Figure 4:**
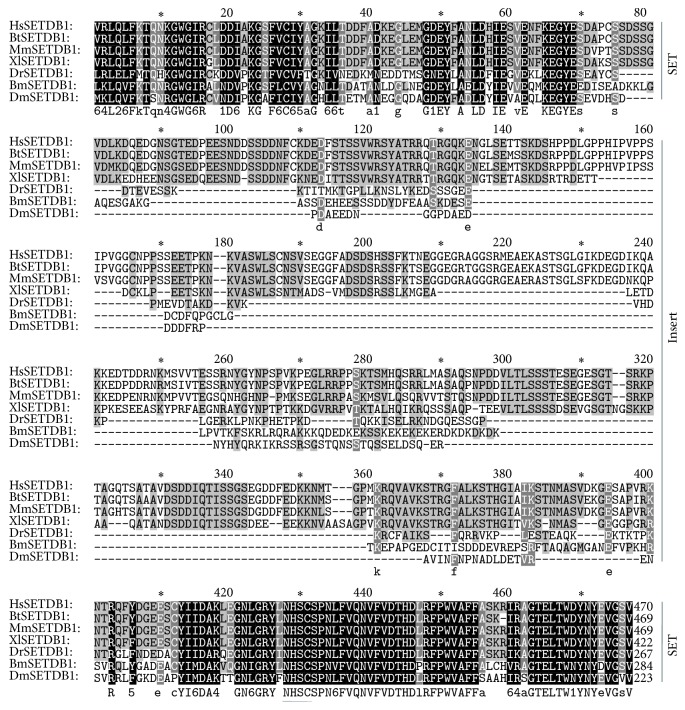
Alignment of SETDB1 SET domains. Hs:* Homo sapiens*; Bt:* Bos taurus*; Mm:* Mus musculus*; Xl:* Xenopus laevis*; Dr:* Danio rerio*; Bm:* Bombyx mori*; Dm:* Drosophila melanogaster*.

SUV39 methyltransferase subfamily functions in specifically methylating H3K9. In mammals, this subfamily consists of six main members, SUV39H1, SUV39H2, G9a, GLP1 (G9a-like protein 1/EuHMT1), SETDB1 (ESET), and CLLL8 (SETDB2). The SET domains of this subfamily are located on the C-terminal of the proteins which are flanked by Pre-SET and Post-SET domain. The Pre-SET domain close to SET domain is a special character of this subfamily's proteins which assists SET domain in carrying out the enzyme activity (Figure 3). We identified three members of this subfamily in silkworm, BmG9a-like, BmSetdb1, and BmSu(var)3-9 (actually, we found two variants of Su(var)3-9 in silkworm; see details in Study of Silkworm SET-Domain Gene BmSu(var)3-9). We have not identified the highly similar homolog proteins of G9a and SETDB2 in silkworm. There is one protein that we name BmG9a-like, because it has very similar domain structure to G9a of other species including ANK (ankyrin repeats) and Pre-SET domain, while Post-SET is replaced by an ALARD domain. Since BmG9a-like shows very low sequence similarity to G9a of other species, it is alone on the phylogenetic tree. Based on all of that, we speculate that BmG9a-like is a unique member existing in silkworm. However, more evidence should be provided. SETDB1 has very special SET domain which is bifurcated by a 100–300 amino acids' insert. We also aligned the SET-domain sequence of BmSetdb1 with other species. SET sequence of SETDB1 is highly between silkworm and other species while the insert sequences differ from one species to another (Figure 4).

#### 3.3.3. SET2 Subfamily

**Figure 5:**
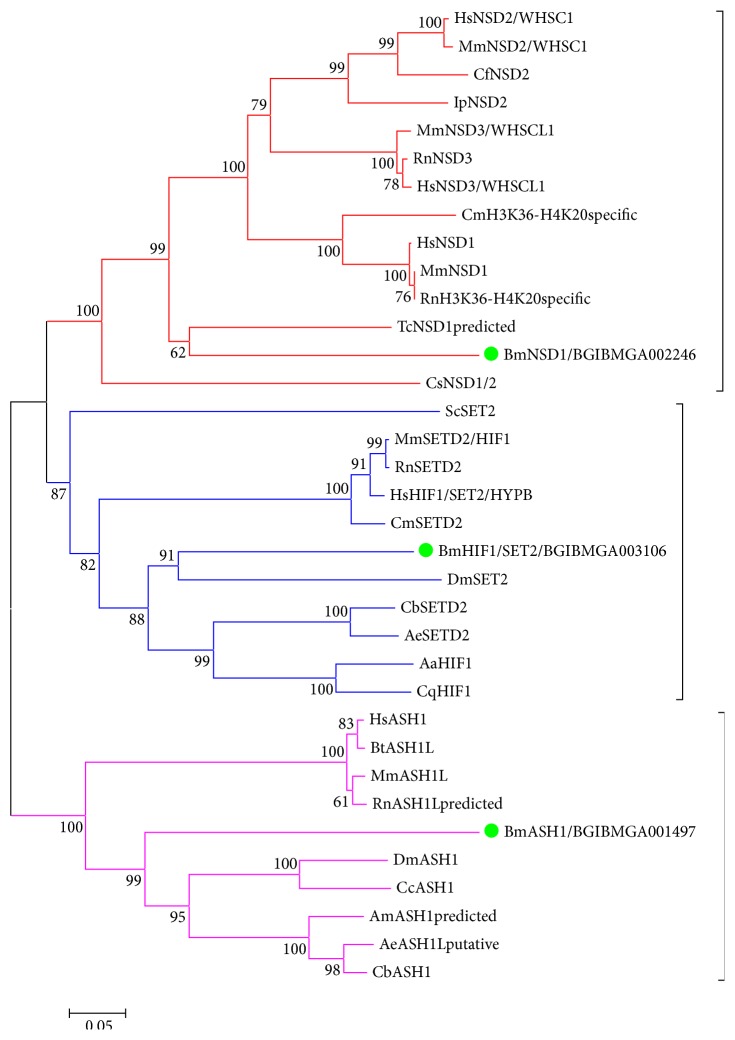
Phylogenetic analysis of SET2 subfamily. Cf:* Camelus ferus*; Ip:* Ictalurus punctatus*; Cm:* Chelonia mydas*; Tc:* Tribolium castaneum*; Cs:* Clonorchis sinensis*; Cb:* Cerapachys biroi*; Ae:* Acromyrmex echinatior*; Aa:* Aedes aegypti*; Cq:* Culex quinquefasciatus*; Cc:* Ceratitis capitata*. The bright green on the phylogenetic tree represents silkworm members (the abbreviations of species mentioned above are omitted here).

SET2 subfamily of mammals mainly includes 5 members, NSD1, NSD2, NSD3, SET2 (HIF1/HYPB), and ASH1. NSD1, NSD2, and NSD3 share the conserved domains such as SET, AWS (Associated With SET), Post-SET, PWWP (a domain containing highly conserved Pro-Trp-Trp-Pro motif), PHD, and Ring finger. In silkworm, we just identified one homolog of the NSD proteins, BmNSD1, which has five of the above mentioned conserved domains except the Ring finger. However, so far, there is no homolog protein of NSD identified in* Drosophila*. HIF1 contains four conserved domains, SET, Post-SET, AWS, and WW. BmHIF1 identified from silkworm database have all domains but the WW domain, which may be because the sequence of BmHIF1 in silkworm is not complete, which is also confirmed by the relatively shorter sequence of BmHIF1 compared to* Drosophila* and other insects. ASH1 has four conserved domains except SET domain, AT hook, BROMO, BAH, and PHD. BmASH1 has all the domains except AT hook. Phylogenetic analysis of SET2 subfamily shows that members of silkworm SET2 subfamily cluster together with their homolog proteins in other species separately (Figure 5).

#### 3.3.4. Expression Profile of Silkworm SET-Domain Genes

**Figure 6:**
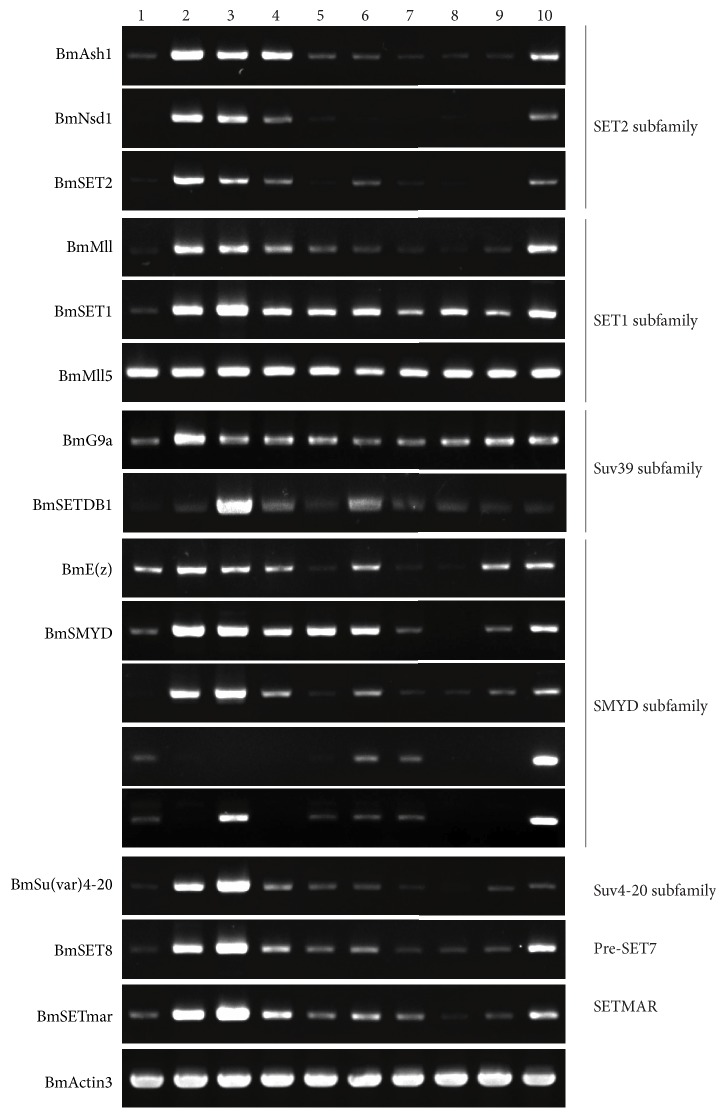
Developmental expression profile of silkworm SET-domain genes.* 1: whole silkworm; 2: ovary; 3: testis; 4: blood; 5: midgut; 6: fat body; 7: body wall; 8: Malpighian tube; 9: silk gland; 10: head*.

The expression profiles of silkworm SET-domain family members show that they have widely high expression level in gonad (testis and ovary) except BGIBMGA002076, which may be related to the gonad's function to propagate the genetic information (Figure 6).

#### 3.3.5. Study of Silkworm SET-Domain Gene BmSu(var)3-9

**Figure 7:**
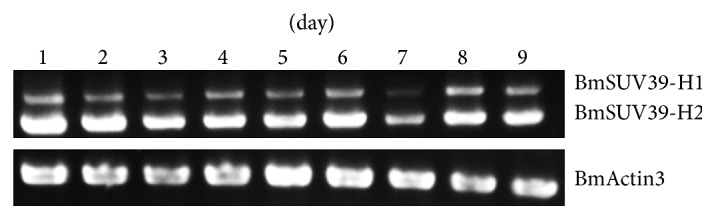
Expression patterns of BmSu(var)3-9 during embryogenesis day 1 to day 9 in silkworm. RT-PCR was performed to detect the expression patterns of BmSu(var)3-9 using specific primers and Actin3 gene was used as the internal control at each time point.

**Figure 8:**
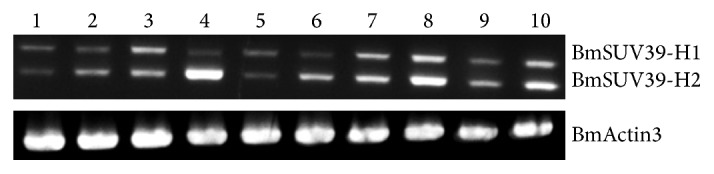
Larval stage 5 day 3 expression profile of BmSu(var)3-9. 1: whole silkworm; 2: ovary; 3: testis; 4: blood; 5: midgut; 6: fat body; 7: body wall; 8: Malpighian tube; 9: silk gland; 10: head.

Through amplification of homolog gene of Su(var)3-9 in silkworm, we found that silkworm has two transcript isoforms. Using RACE technology to obtain the full-length cDNA sequence showed that the situation is different from other species; the two transcript isoforms have a different sequence of 846 bp at the 5′ end which belongs to the 5UTR of the longer spliceosome, and they encode a protein of 317 and 593 amino acids separately. Embryonic expression analysis of different period and different organs of three-day, fifth-instar larvae of silkworm displays that Su(var)3-9 is highly expressed during the 1–9 days of development of embryo, whereas it is relatively low expressed in the larvae organs (Figures 7 and 8).

## 4. Conclusion

SET-domain superfamily is a group of histone lysine methyltransferases which form different methylation marks (mono-, di-, and trimethylation, also known as me1, me2, and me3) on histone by transferring the methyl from S-adenosyl-L-methionine (AdoMet) to lysine residues of histone proteins, with production of S-adenosyl-L-homocysteine (AdoHcy). The core enzymatic sites of the methyltransferases are located in the SET domain. Since the first SET-domain protein* Drosophila* DmSu(var)3-9 had been identified at the end of the last century, researchers confirmed the methyltransferase activity of Su(var)3-9 protein in mammals for the first time at the beginning of the 20th century. SET-domain proteins are widely identified from the lower eukaryote yeast to higher human. Human has more than 100 SET-domain-containing proteins. The great SET-domain family is linked to its great function in epigenetic regulation. SET-domain-containing proteins play important roles in the development process of human,* Drosophila*, and* Arabidopsis*. There are very few reports on the study of histone methylation in silkworm. Currently, the only one SET-domain protein reported is BmE(z) which is the main member of PcG (Polycomb group genes) proteins. The methylation of H3K27 by BmE(z) has been confirmed in silkworm by knocking down BmE(z) which leads to the reduction of H3K27me3 level [18]. Besides, demethyltransferase has been reported in silkworm, BmLid. The function of demethylating H3K4me2, H3K4me3, H3K9me2, H3K9me3, and H3K27me3 of BmLid has been validated and it is supposed to have wider catalytic substrate than its homolog protein in mammals and* Drosophila* [19].

In our study, from the silkworm database, 24 genes of SET-domain family were identified and 17 out of them were included in SET1, SET2, SUV39, SUV4-20, EZ, and SMYD and six subfamilies. Except for three from SUV39 subfamily members, four from SET1 subfamily members, three from SET2 subfamily members, one from SUV4-20 subfamily members, one from EZ subfamily members, and five from SMYD subfamily members, the remaining five were independent members and two out of them were the homologous genes. Although the RIZ subfamily had not been identified in the silkworm, the certain members had already covered the common members of other species. Comparative analysis for identifying the SET-domain family members showed that they were highly conserved in each species, including the substrate binding site and catalytic site. Evolutionary analysis of the representative subfamily showed that the structure of the SUV4-20 family was highly consistent in the large number of species including the silkworm. Identified in the silkworm,* Bombyx mori* SUV39 subfamily members SETDB1 and su(VaR)3-9 domain composition are highly conserved among various species, especially SETDB1 unique for sequence insertion and splitting the set domain in the silkworm,* Bombyx mori* and other species are extremely conservative. In addition, G9a-like gene BGIBMGA007949 in the silkworm possessed the conservative composition and structure of G9a and group specificity. BGIBMGA001497, BGIBMGA003106, and BGIBMGA002246 in the silkworm were three respective homologous genes of SET2 subfamily. Interestingly, Su(VaR)3-9 in embryos from day 1 to day 9 and third day of the fifth larval stage were highly expressed, suggesting an important role possibly in the embryonic differentiation process.

In summary, based on the silkworm genome database, we identified, cloned, and analyzed the SET-domains proteins in silkworm,* Bombyx mori*. We intend to study the common and unique features of silkworm SET-domain family members, which will provide more information and important reference to the whole SET-domain proteins.

## Figures and Tables

**Table 1:** SET-domain genes identified from the SilkDB.

Subfamily	Gene name	Gene holoname	Name in SilkDB	Conserved domain (addition to SET)	ORF length (bp)	Chromosome number	Scaffold	ESTs	Probe	*E*-value	Methylase activity in other species
SUV39	*BmG9a-like/Ehmt2*	Euchromatic histone-lysine N-methyltransferase 2	BGIBMGA007949	Ankyrin repeats, Pre-SET, and ALAD	4161	15	nscaf2888	7	sw00703, sw17591, sw15556, and sw17724	3*e* − 34	H3K9, H3K27
	*BmSetdb1*	SET-domain, bifurcated 1	BGIBMGA011407	Tudor, MBD, Pre-SET, and Post-SET	2895	23	Nscaf3026	No	sw21024, sw18443	0.0	H3K9
	*BmSu(var)3-9*	Suppressor of variegation 3-9	BGIBMGA011500, BGIBMGA011499	CHROMO, Pre-SET, and Post-SET	1782	23	Nscaf3027	12	sw11427, sw04471	0.0	H3K9

SET1	*BmMll*	Myeloid/lymphoid or mixed-lineage leukemia	BGIBMGA010221	PHD, FYRN, FYRC, and Post-SET	12657	7	nscaf2986	2	sw12266, sw14974	0.0	H3K4
	*BmSet1*	SET-domain-containing 1	BGIBMGA012978	Post-SET	4080	16	Nscaf3058	No	sw14237, sw14238, sw16463, and sw04801	0.0	H3K4
	*BmTrr*	Trithorax-related	BGIBMGA002503, BGIBMGA002502	PHD, FYRN, and Post-SET	—	9	nscaf2511	4	sw05846, sw04238, sw06661, and sw10095	0.0 4*e* − 156	H3K4
	*BmMll5*	Myeloid/lymphoid or mixed-lineage leukemia 5	BGIBMGA004310	PHD		20	nscaf2789	No	sw00989, sw16148	2*e* − 27	H3K4
	*BmSET2*	SET-domain-containing 2	BGIBMGA003106	AWS, Post-SET	3633	4	nscaf2589	No	sw11671, sw11672	2*e* − 156	H3K36

SET2	*BmNsd1*	Nuclear receptor binding SET-domain protein 1	BGIBMGA002246	PWWP, PHD, AWS, and Post-SET	6600	26	nscaf2330	3	sw11742	4*e* − 153	H3K36, H4K20
	*BmAsh1*	Absent, small, or homeotic discs 1	BGIBMGA001497	AWS, Post-SET, BROMO, PHD, and BAH	8754	21	nscaf2136	2	sw11562	0.0	H3K4, H3K9, and H4K20

SUV4-20	*BmSu(var)4-20*	Suppressor of variegation 4-20	BGIBMGA000919	—	2094	13	Nscaf1898	4	sw13536	2*e* − 142	H4K20

EZ	*BmE(z)*	Enhancer of zeste	BGIBMGA014476	SANT, CxC		—	Scaffold707	6	sw15702, sw16217	0.0	H3K27

SMYD			BGIBMGA014048	zf-MYND	1782	—	Nscaf3115	1	sw10812	3*e* − 78	—
			BGIBMGA007907	—	1881	15	nscaf2888	No	sw11109	2*e* − 128	—
			BGIBMGA002076	—	2232	1	nscaf2210	1	sw19609	1*e* − 86	—
			BGIBMGA011073	—	1155	23	Nscaf3015	No	sw20741	1*e* − 139	—
			BGIBMGA002109	—	1071	1	nscaf2210	No	sw18112, sw02496	2*e* − 48	—
			BGIBMGA008839	zf-MYND		3	nscaf2927	No	sw17374, sw17507	4*e* − 59	—
			BGIBMGA008939	zf-MYND		3	nscaf2930	No	sw08614	2*e* − 77	—
			BGIBMGA008838	—		3	nscaf2927	No	sw21409	2*e* − 92	—
			BGIBMGA008923	—		3	nscaf2930	No	sw21256	2*e* − 164	—
			BGIBMGA003139	—		4	nscaf2589	No	sw05603	1*e* − 68	—

Others	*BmSet8/PR-set7*	SET-domain-containing (lysine methyltransferase) 8	BGIBMGA012853	—	912	16	Nscaf3058	1	—	3*e* − 76	H4K20
	*BmSetmar*	SET-domain and mariner transposase fusion gene	BGIBMGA007557	—	810	15	nscaf2887	2	—	2*e* − 53	—
